# Optimal fetal growth for the Caucasian singleton and assessment of appropriateness of fetal growth: an analysis of a total population perinatal database

**DOI:** 10.1186/1471-2431-5-13

**Published:** 2005-05-24

**Authors:** Eve M Blair, Yingxin Liu, Nicholas H de Klerk, David M Lawrence

**Affiliations:** 1 Centre for Child Health Research, The University of Western Australia, at the Telethon Institute for Child Health Research, P.O. Box 855, West Perth. WA. 6872, Australia; 2 Centre for Developmental Health, Curtin University of Technology and Telethon Institute for Child Health Research, P.O. Box 855, West Perth. WA. 6872, Australia

## Abstract

**Background:**

The appropriateness of an individual's intra uterine growth is now considered an important determinant of both short and long term outcomes, yet currently used measures have several shortcomings. This study demonstrates a method of assessing appropriateness of intrauterine growth based on the estimation of each individual's optimal newborn dimensions from routinely available perinatal data. Appropriateness of growth can then be inferred from the ratio of the value of the observed dimension to that of the optimal dimension.

**Methods:**

Fractional polynomial regression models including terms for non-pathological determinants of fetal size (gestational duration, fetal gender and maternal height, age and parity) were used to predict birth weight, birth length and head circumference from a population without any major risk factors for sub-optimal intra-uterine growth. This population was selected from a total population of all singleton, Caucasian births in Western Australia 1998–2002. Births were excluded if the pregnancy was exposed to factors known to influence fetal growth pathologically. The values predicted by these models were treated as the optimal values, given infant gender, gestational age, maternal height, parity, and age.

**Results:**

The selected sample (N = 62,746) comprised 60.5% of the total Caucasian singleton birth cohort. Equations are presented that predict optimal birth weight, birth length and head circumference given gestational duration, fetal gender, maternal height, age and parity. The best fitting models explained 40.5% of variance for birth weight, 32.2% for birth length, and 25.2% for head circumference at birth.

**Conclusion:**

Proportion of optimal birth weight (length or head circumference) provides a method of assessing appropriateness of intrauterine growth that is less dependent on the health of the reference population or the quality of their morphometric data than is percentile position on a birth weight distribution.

## Background

Being born small for one's gestational age is associated with adverse outcomes in both the short and long term [1-3]. However assessing whether a neonate is at risk of compromise on account of inappropriate intrauterine growth is complicated because not all fetuses *should *grow at the same rate [4]. Currently the appropriateness of fetal growth is usually inferred from the percentile position that the neonate's birth weight occupies on a gestation-specific birth weight distribution, that may also be specific for gender. This practice is unsuitable if the most appropriate birth weight for the neonate being assessed is not the same as that for all members of the population contributing to the distribution. Additional problems associated with percentile-based standards are (a) the implications of a given percentile position vary with the burden of growth restricting pathology in the source population, (b) the estimation of percentile position is imprecise at the extremes of a distribution where the information is of most clinical importance and (c) since percentile positions represent an ordinal rather than interval or ratio scale, the possibilities for valid statistical manipulation are limited [5].

This communication seeks to describe, demonstrate and justify the usefulness of an alternative method of assessing the appropriateness of fetal growth from information available in the neonatal record.

This method is based on three underlying concepts:

1. Appropriateness of growth can be expressed as the ratio of the observed birth dimension to the optimal birth dimension for that an individual neonate. Considering the dimension of weight we refer to this ratio as the proportion of optimal birth weight (POBW): a concept similar to the birth weight ratio [6]. Assessing appropriateness of growth then requires values for the optimal birth dimensions for the neonate in question.

2. Optimal intrauterine growth is most likely to be achieved during pregnancies unaffected by any maternal or fetal pathology or exposures that can pathologically affect fetal growth,

3. The many determinants of fetal growth can be classified as having either a pathological or a non-pathological effect on growth.

Factors with non-pathological effects on growth include gestational duration, gender [7-9], maternal size [10] and parity [7,11] and paternal size [12]. We define optimal birth weight as that achieved when no factors are present that can exert a pathological effect on growth. The central tendency of the distribution of birth weights in a population which experiences no factors that exert a pathological effect on intrauterine growth is taken as the optimal birth weight for neonates with the same combination of non-pathological determinants of fetal growth.

Pathological growth determining factors, such as maternal vascular disease or those associated with congenital malformations, usually restrict fetal growth. More rarely fetal weight is pathologically increased, the most well known example being fetal macrosomia induced by maternal diabetes [13].

It is less useful to categorise as either *pathological *or *non-pathological *those determinants of growth that cannot easily be altered. Multiple pregnancy and maternal race are two examples of such determinants.

This paper demonstrates our method of assessing the appropriateness of fetal growth by deriving equations for optimal birth weight, birth length and head circumference. Gestational duration, fetal gender and maternal height, age and parity are considered as potential independent variables representing the non pathological determinants of fetal growth. In order to select a population with optimal intrauterine growth, all births with evidence of having been exposed to pathological determinants of fetal growth are excluded. Appropriateness of fetal growth is then expressed as the ratio of the observed birth dimension to the estimated optimal birth dimension for a neonate with the same values for non-pathological determinants of fetal growth. The utility of this approach is discussed.

## Methods

### Sample selection

Records of the 126,393 births in Western Australia (WA) during the period of 1998 to 2002 were obtained from the Western Australian Maternal and Child Health Research Database (MCHRDB) [14]. This period was selected because data concerning whether the mother smoked during pregnancy, the most prevalent environmental exposure with a pathological effect on intrauterine growth, are available on this data base for births from 1998 onwards. The most recent available cohort at the time of writing was 2002.

Of 1998–2002 WA births, 85% were to Caucasian women and 96.8% were singletons. This example therefore derives standards for singletons born to Caucasian women, see Discussion for generalisability.

To achieve a cohort of Caucasian singletons anticipated to exhibit optimal fetal growth, any pregnancy with evidence to suggest that fetal growth may have been affected pathologically must be excluded. Stillbirths and deaths before 28 days were excluded as evidence of a suboptimal intrauterine course, which may be associated with abnormal growth, and, for stillbirths, because duration of intrauterine growth, as opposed to gestational duration to delivery, is not recorded.

The selection of further exclusion criteria was guided, in part, by the extensive literature concerning growth restriction as reviewed by Resnik [15]. Suggested exclusion criteria for which data are available in the MCHRDB are listed in Table 1 in order of their frequency observed in 1998–2002 WA births. Resnik [15] also suggests that maternal gestational use of anticonvulsants, cocaine, heroin or alcohol, maternal thrombophilic disorders and nutritional deprivation are risk factors for growth restriction. While these variables are not available on the MCHRDB, 0.5% of 1998–2002 WA mothers were recorded as having epilepsy and may have been on anticonvulsants. The use of cocaine and heroin are illegal in WA and very likely to be under-reported in medical records. Their use, along with that of excess alcohol, is associated with birth defects. Since both a birth defect and death before 28 days are exclusion criteria, it is anticipated that the majority of births significantly affected by these substances will be excluded. The incidence of thrombophilic disorders varies with ethnic background and no data are available concerning its frequency in WA pregnant women who are of mixed ethnic backgrounds. However neither thrombophilic disorders nor macro-nutrient deprivation are noted as problems in the WA pregnant population. Thus the factors listed in Table 1 are anticipated to represent the most frequently occurring pathological determinants of fetal growth in our population and were excluded from the sample for the purposes of deriving measures of optimal fetal growth. Socio-demographic variables of the selected sample were compared with those of excluded Caucasian singletons.

**Table 1:** Observed frequency of factors known to be associated with pathological deviations in fetal growth: All Western Australian births 1998–2002.

**Factor**	**N**	**%**
Maternal Smoking	27,326	21.62
Maternal vascular disease	8,334	6.59
Birth defects [29]	7,520	5.95
Maternal (pre-existing or gestational) diabetes	5,051	4.00
Multiple pregnancy	3,991	3.16
TORCH infections^a^	2,945	2.33
High altitude^b^	0	0.0

a) Toxoplasmosis, Rubella, CMV, Herpes

b) No population centre in Western Australia is above 300 m. The highest peak (Mt. Meharry) is 1253 m, and like all Western Australian peaks, is situated in an unpopulated area.

### Gestational age data

Since the primary determinant of birth dimensions is the duration of growth, reliable estimates of gestational duration (GA) are essential, yet exclusion criteria for poor quality gestational data are likely to exclude a biased sample. Details of the algorithm used to obtain the best estimate of GA from all available data are described and justified elsewhere [16]. Applying this method to the total 1998–2002 WA birth population resulted in no satisfactory gestational estimate being available for only 97 births (~0.1%) and being beyond the range of 23–42 weeks for a further 573 subjects. Birth weight and gestational duration data for remaining births were examined to exclude combinations so unlikely as to suggest error in the gestational datum. The cut off birth weights at each gestational age between 23 and 36 weeks, above which the observation was excluded, were selected with a view to excluding infants at least four gestational weeks older than reported, since break through bleeding at four-week intervals in early pregnancy is a source of gestational error in women claiming to be certain of the date of their last menstrual period. Due to the slower rate of weight accretion with respect to weight dispersion in infants born at term, this method of data cleaning is not applicable for births reported as being at greater than 36 weeks gestational duration [17].

### Analysis

For each of the three response variables (birth weight, length and head circumference) the Box-Cox transformation [18] was used to identify the optimal transformation to reduce non-normality and heteroscedasticity of errors. Fractional polynomial regression was then used to identify the best fit transformation of gestational age to account for any non-linearity in the relationship between gestational duration and each response variable [19]. Fractional polynomials are a means of identifying the curve of best fit in cases where non-linearity is possible but there is no scientific reason to specify the shape of the non-linear relationship. Royston and Altman claim that their set of power transformations have the flexibility to cover almost all likely shapes of non-linear relationship. The number of possible inflection points is determined by the order of the fractional polynomials fitted. In this case, where a sideways "S" shape is expected, 2nd order fractional polynomials, which allow for up to two inflection points, are sufficient. To aid computation, gestational duration was included in the fractional polynomial regressions as GA/100.

Maternal height (cm) and maternal age (years) were included as linear predictor variables (see Discussion) centred on the population mean values of 162 cm and 25 years respectively. Infant sex and maternal parity were included as categorical variables. Parity was categorised with first birth as the reference, second and third birth as two separate categories, and fourth and subsequent births constituting the fourth category. Models were fitted using SAS (Version 8.2) (SAS Institute Inc., 2001). The fit of each model was tested by plotting residuals against GA and against the predicted dependent variable (weight, length or head circumference at birth). Additionally, POBWs for 3^rd^, 10^th ^and 90^th ^percentile birth weight were estimated within each completed gestational week for sub-samples of parity and maternal height, to ensure that the model adjusted appropriately for these non-pathological determinants. Finally, to aid clinical interpretation of POBW values, POBW was estimated for the 3^rd^, 10^th ^and 90^th ^percentile positions by taking the weighted mean of POBWs estimated for each parity/gender stratum of births between 38 and 41 gestational weeks inclusive, assuming a constant maternal height of 162 cm.

## Results

Table 2 lists the numbers of births sequentially excluded by each exclusion criterion, and shows that 62,746 singleton Caucasian births remained for analysis. Equations for optimal growth were derived from this total population sample of singleton, Caucasian births without recognised risk factors for growth abnormality, which comprised 49.7% of all Western Australian births and 60.5% of the Caucasian singleton births. Table 3 compares the distributions of socio-demographic variables for included births with those of excluded Caucasian singleton births. As anticipated, given the sample size and the selection criteria, the difference in all distributions is statistically very significant, with the exception of gender, which is nonetheless significantly different at the p = .05 level. However the clinical differences tend to be small with the exception of the proportion of preterm and very preterm births.

**Table 2:** Sample selection: the number of births sequentially excluded by each exclusion criterion.

Exclusion Criterion	Number (%) excluded	Number remaining
All WA births 1998–2002	0	126,393
Not Caucasian	18,968 (15.0)	107,425
Multiple pregnancy	3,532 (3.3)	103,893
Stillbirth	606 (0.6)	103,287
Maternal gestational smoking	21,570 (20.9)	81,717
Growth restricting conditions^a^	9,970 (12.2)	71,747
Gestation <23 or >42 weeks	146 (0.2)	71,601
Birth weight excessive for GA	75 (0.1)	71,526
Missing essential variable^b^	5,254 (7.3)	66,272
Death before 28 days	102 (0.2)	66,170
Birth defect^c^	3,424 (5.2)	62,746

a. as identified in Table 1.

b. Essential variables were maternal height and age, birth weight, length and head circumference: almost all exclusions at this stage were the result of missing values for maternal height.

c. [28]

**Table 3:** Comparison of distributions of selected characteristics among Caucasian singleton births which were or were not included in the study.

	Included	Excluded*	
Denominator, N	62,747	maximum 41,146	
**Sex**			p
Male fetus, % (N)	50.62 (31,760)	51.63 (21,242)	0.0014
**Gestational age**			
Mean GA (sd), wks	39.0 (1.6)	38.2 (2.6)	<.0001
GA<37 weeks, % (N)	4.55 (2,856)	9.96 (4,099)	<.0001
GA<33 weeks, % (N)	0.53 (334)	2.65 (1,092)	<.0001
5^th^-95^th ^percentile, wks	37–41	35–41	
**Birth weight**			
Mean weight (sd), g.		3,282 (641)	<.0001
5^th^-95^th ^percentile, g	2695–4270	2265–4170	
**Birth length**			
Mean length (sd), cm	50.4 (2.5)	49.5 (3.7)	<.0001
5^th^-95^th ^percentile, cm	46–54	45–54	
**Birth head circumference**			
Mean circumference, cm	34.7 (1.6)	34.2 (2.4)	<.0001
5^th^-95^th ^percentile, cm	32–37	31–37	
**Maternal characteristics**			
Mean height, cm	165.1 (6.7)	164.7 (6.7)	<.0001
5^th^-95^th ^percentile, cm	154–176	153–176	
Mean age, y	29.4 (5.2)	28.5 (5.7)	<.0001
5^th^-95^th ^percentile, y	20–38	19–38	
Socio-economic Disadvantage†	1009.3 (78.8)	984.7 (86.0)	<.0001

* Variable denominators as a result of missing values.

† [29]

### Birth weight

The Box-Cox procedure suggested that square root was the optimal transformation to use for normalising birth weight. The optimal fractional polynomial gestational age terms were GA^3 ^and GA^3^ln(GA). In multivariate analysis, maternal age was not a significant predictor of birth weight. Parameter estimates for the selected best fitting regression equations for the square root of birth weight are given in Table 4. This model has an adjusted R^2 ^of 40.5%. The best fitting regression equation for estimating optimal birth weight (grams) can therefore be expressed as:

**Table 4:** Parameter estimates modelling the square root of birth weight (grams)

**Independent Variable**	**Parameter Estimate**	**Standard Error**	**t value Pr >|t|**	**95% confidence limits**	
**Intercept**	-14.08	0.73	-19.36 <.0001	-15.51	-12.66
*GA*^3 ^*	-1413.6	25.0	-56.43 <.0001	-1463	-1365
*GA*^3 ^In (*GA*^3^) *	-2782.5	39.4	-70.55 <.0001	-2860	-2705
**Male infant**	1.185	0.027	44.22 <.0001	1.13	1.24
**Maternal height cm#**	0.1077	0.0024	45.63 <.0001	0.103	0.112
**2nd birth**	1.0277	0.031	33.40 <.0001	0.967	1.088
**3rd birth**	1.318	0.0399	33.04 <.0001	1.24	1.40
**4th and subsequent birth**	1.571	0.054	29.33 <.0001	1.46	1.68
**GA# × Maternal height**	0.00667	0.00123	5.42 <.0001	0.0043	0.0091
**Adjusted R^2 ^for this model is 0.405**					

* indicates non-centred but scaled GA, divided by 100

# indicates a centred variable – GA centred around mean of 40 weeks, maternal height centres around a mean of 162 cm



**Figure 1:**
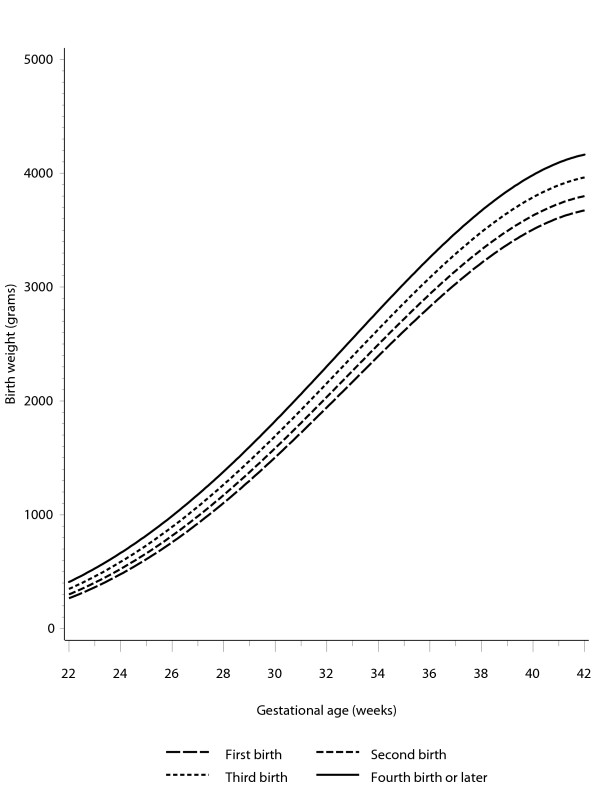
Mean of male and female optimal birth weight by gestational age at delivery and parity, estimated for births to women of height 162 cm.

**Figure 2:**
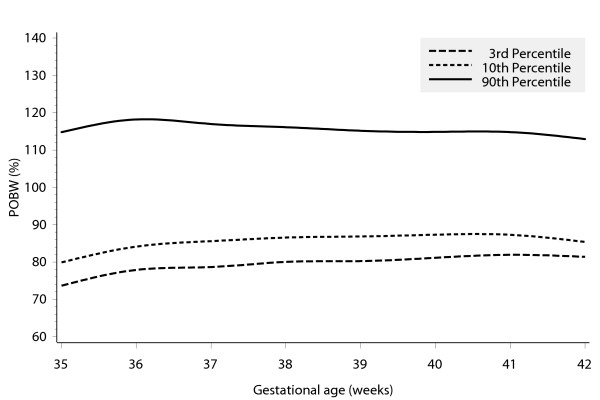
Weighted mean POBW (across 8 parity/gender combinations) observed at the 3rd, 10th and 90th percentile positions on the birth weight distributions, by gestational age at delivery.

and, of course,



This equation suggests that under our standard conditions of birth at 40 weeks gestation to a 162 cm primiparous woman, female infants should weigh 3436.0 g and males 3576.4 g. Second births should weigh 123 g more, third births 158 g more and fourth or subsequent births 189 g more than the first birth. An example of the curves obtained from this equation is shown in Figure 1. The weighted mean POBWs (across the 8 parity/gender combinations) observed at the 3^rd^, 10^th ^and 90^th ^percentile positions on the birth weight distribution are shown by gestational duration across the range 35–42 weeks in Figure 2. These ratios change little by gestational duration within this range. The weighted mean POBWs across the range 38–41 weeks for the 3^rd^, 10^th ^and 90^th ^percentile birth weights are 81%, 87% and 115% respectively, Table 7.

### Birth length

The Box-Cox procedure suggested that birth length raised to the power of 0.75 was the optimal transformation to use for normalising birth length. The optimal fractional polynomial gestational age terms were GA^2 ^and GA^3^. In multivariate analysis, maternal age was not a significant predictor of birth length. Parameter estimates for the selected best fitting regression equations for the square root of birth weight are given in Table 5. This model has an adjusted R^2 ^of 32.2%. The best fitting regression equation for estimating optimal birth weight (grams) can therefore be expressed as:

**Table 5:** Parameter estimates modelling birth crown heel length to the power of 0.75 (cm)

**Independent Variable**	**Parameter Estimate**	**Standard Error**	**t value Pr >|t|**	**95% confidence limits**	
**Intercept**	5.684	0.176	32.30 <.0001	5.34	6.03
Scaled gestational age squared*	209.9	3.66	57.31 <.0001	203	217
Scaled gestational age cubed*	-318.5	6.44	-49.43 <.0001	-331	-305
**Male infant**	0.2350	0.0047	50.08 <.0001	0.226	0.244
**Maternal height c#**	0.01665	0.00042	40.28 <.0001	0.0158	0.0174
**2nd birth**	0.07484	0.0054	13.89 <.0001	0.064	0.085
**3rd birth**	0.1161	0.0070	16.62 <.0001	0.102	0.130
**4th and subsequent birth**	0.1508	0.0094	16.09 <.0001	0.132	0.169
**GA# × Maternal height**	0.000763	0.000215	3.54 0.0004	0.00034	0.00120
**Adjusted R^2 ^for this model is 0.322**					

* indicates non-centred but scaled GA, divided by 100

# indicates a centred variable – GA centred around mean of 40 weeks, maternal height centres around a mean of 162 cm



and of course



This equation suggests that under our standard conditions females should be 50.3 cm long at birth and males should be 0.83 cm longer. The weighted mean proportions of optimal crown heel length at 3^rd^, 10^th ^and 90^th ^percentile positions of crown heel length were found to be 93%, 95% and 105% respectively, see Table 7.

### Birth head circumference

The Box-Cox procedure suggested that it was neither necessary nor desirable to transform head circumference prior to modelling. The optimal fractional polynomial gestational age terms were GA and GAln(GA). All potential predictor variables significantly predicted head circumference, including maternal age. Parameter estimates for the selected best fitting regression equations for the square root of birth weight are given in Table 6. This model has an adjusted R^2 ^of 25.2%. The best fitting regression equation for estimating optimal birth weight (grams) can therefore be expressed as:

**Table 6:** Parameter estimates modelling head circumference at birth (cm)

**Independent Variable**	**Parameter Estimate**	**Standard Error**	**t value Pr >|t|**	**95% confidence limits**	
**Intercept**	-88.31	1.92	-46.31 <.0001	-92.04	-84.57
Scaled gestational age*	43.31	0.38	115.06 <.0001	42.57	44.05
GA ln (GA) *	-287.6	5.1	-56.32 <.0001	-297.6	-277.6
**Male infant**	0.6072	0.0109	55.89 <.0001	0.5860	0.6285
**Maternal height cm #**	0.02745	0.000956	28.68 <.0001	0.0256	0.0293
**2nd birth**	0.2352	0.0127	18.52 <.0001	0.210	0.260
**3rd birth**	0.3151	0.0167	18.92 <.0001	0.282	0.348
**4th and subsequent birth**	0.3394	0.0225	15.09 <.0001	0.295	0.384
**Maternal age #**	0.01322	0.00110	12.01 <.0001	0.0111	0.0154
**GA# × Maternal height**	0.00107	0.000498	2.14 0.0321	0.000091	0.00205
**Adjusted R^2 ^for this model is 0.252**					

* indicates non-centred but scaled GA, divided by 100

# indicates a centred variable – GA centred around mean of 40 weeks, maternal height is centred around a mean of 162 cm, maternal age is centred around a mean of 25 years

**Table 7:** Percentage of optimal birth dimension equivalences of percentile cut points from which appropriateness of growth has traditionally been inferred: as observed in this sample of optimally grown neonates.

	**Percentage of optimal birth dimension (%)**		
Percentile Position	Weight	Length	Head circumference
3^rd^	80	93	93
10^th^	87	95	96
90^th^	115	105	105



This equation suggest that under our standard conditions and for mothers of 25 years, the optimal head circumference for females was 34.4 cm and for males, 0.61 cm larger. The weighted mean proportions of optimal head circumference at 3^rd^, 10^th ^and 90^th ^percentile positions of head circumference were found to be 93%, 96% and 105% respectively, see Table 7.

## Discussion

These regression equations, derived from a population based sample of more than 62,000 singleton Caucasian pregnancies without the major risk factors for intrauterine growth anomaly, demonstrate the method used at our Institute to assess appropriateness of fetal growth. The method is applicable to all populations with suitable data available. Our results may be directly applicable to other populations besides Western Australian Caucasian and Aboriginal singletons, and we consider it likely to be applicable to all Caucasian populations, but applicability should be verified as suggested below.

### Advantages of the ratio method

The use of ratios, such as POBW, in the measurement of intrauterine growth is not a novel idea, [6,20] but has not been universally adopted despite the many advantages of ratios over the more commonly used percentile positions. The following discussion applies to all ratios of optimal dimensions, but, for simplicity POBW will be used as the example throughout. These advantages are:

a) Ratios, such as POBW, represent continuous interval measures.

b) Estimations of POBW require only a single standard value, the predicted optimal birth weight, rather than values at several points on the birth weight distribution. The precision of estimating a percentile position varies inversely with observation density and, since the majority of distributions have fewer observations at the extremes, extreme observations will be the least precise, whatever the size of the sample generating the distribution. Extreme observations are also most subject to error. When verification of individual observations is not possible (as with de-identified data), it is a common practice to exclude extreme values on the assumption that they *are *in error, significantly altering the estimated value of extreme percentile positions. The positions of percentile extremes are therefore both imprecise and sensitive to actual and perceived data quality. The most precise percentile estimates are those at the highest observation densities, which, since many distributions are akin to Gaussian (particularly those of birth dimensions), is often the 50^th ^percentile or median[5].

c) Births affected by growth disturbing factors are over-represented in the extremes of the growth distribution. Hence the positions of extreme percentiles are sensitive to the incidence of growth affecting pathologies in the reference population and vary with the health of the reference population. For example, a newborn with a POBW of 85% might be at the 20^th ^percentile position of the birth weight distribution for a population with a high burden of growth restricting pathologies, but the 8^th ^percentile of a population with optimal fetal growth. For an extreme percentile position to be meaningful therefore, the health status of the population from which it is derived needs to be defined, whereas the predicted birth weight is less sensitive to disease burden. Though less sensitive, the proportion of the reference population with growth disturbing factors will also affect the predicted birth weight, except in the unlikely situation where pathological restriction is balanced by pathological acceleration. For this reason we sought to identify a population without growth disturbing factors. The ratio of observed birth weight to predicted birth weight is more generalisable than extreme percentile positions, and the ratio of observed to predicted *optimal *birth weight is even more generalisable[5].

d) POBW is a continuous scale that correlates with weight deficit, whereas percentile position is an ordinal scale that does not. For example, Table 8 considers a population sample with a normal (Gaussian) birth weight distribution, mean birth weight of 3,400 g and standard deviation of 345 g. Being Gaussian, the predicted weight equals the mean (and 50^th ^percentile position) or 3,400 g. In Table 8, changes in percentile position of 4 or 5 percentile points are shown to represent changes in weight of between 43 g and 151 g depending on the particular percentile positions, whereas, within a population, there is a linear correlation between differences in weight and change in POBW. Equivalent changes in percentile position do not represent equivalent changes in weight. Furthermore, in a total population the presence of growth restricting factors creates a negatively skewed (non-Gaussian) birth weight distribution, so the observed range of birth weights covered by extreme percentiles is broader than indicated in Table 8 and is unpredictable.

**Table 8:** Comparison of changes in percentile position and in POBW for selected changes in birth weight, for a neonate with an estimated optimal birth weight of 3,400 g.

Percentile positions (percentile points)		Change in birth weight (grams)		Change in POBW (%)	
Range	Change	Range	Change	Range	Change
50^th^-45^th^	5	3400-3357	43	100-98.74	1.3
10^th^-5^th^	5	2958-2832	126	87.0-83.3	3.7
5^th^-1^st^	4	2832-2681	151	83.3-78.9	4.4

Failure to utilise the advantages of a ratio may in part be due to clinical unfamiliarity. In contrast to percentile position, there is little literature describing the clinical associations of appropriateness of growth expressed as proportions of a desirable birth dimension [21]. For this reason we have included Table 7 which gives the estimated mean, over gestational weeks 38 – 41 inclusive and each gender and parity group, proportion of optimal ratio values of the 3^rd^, 10^th ^and 90^th ^percentile positions, of each distribution of weight, length and head circumference at birth. This table of equivalences enables an approximate *translation *of the literature using percentile positions to percentages of optimal dimensions. The populations from which percentiles are derived will seldom be confined to those without factors affecting growth pathologically. The proportion of optimal equivalences in Table 7 will over-estimate the appropriateness of growth of percentile defined groups to an extent depending on the burden of growth restricting pathologies in their reference sample. When POBW becomes familiar, its numerical values will convey more precise and generalisable clinical meaning than the traditional percentile positions.

### Sample selection

The sample was limited to singleton births to Caucasian mothers. It is not useful to classify all determinants of intrauterine growth according to whether or not they have a pathological effect on growth. For example, twin pregnancy slows fetal growth particularly in the third trimester; and gestation-specific perinatal outcomes for multiple births delivered at term are not as good as those for singletons [22-24]. However, it is seldom desirable to reduce twin pregnancies to singleton pregnancies and reasonable to ask whether a twin fetus is growing appropriately, given that it is a twin. Multiplicity-specific fetal growth standards would be required to answer this question.

Maternal race is also a problematic factor. The observed variation in intra-uterine growth rates between ethnic groups [7,6] may reflect genetically determined differences in optimal rates and/or systematic differences in incidence of growth restricting pathologies and/or environmental exposures. That is, the association between growth and maternal race may arise as a result of either or both non-pathological and pathological determinants of fetal growth. If racial variation in intrauterine growth arises purely as a result of pathological determinants, maternal race is merely associated with growth rate, rather than being a determinant, and should not be controlled. The balance between non-pathological and pathological influences is likely to vary between ethnic groups and between locations. For example, in Western Australian (WA) Indigenous communities the tendency to slower fetal growth relative to Caucasians is believed to be primarily a result of a higher incidence of growth restricting pathologies and environmental exposures [11]. In south east Asian communities living in WA women also tend to have small babies but their perinatal outcomes are similar to those of WA Caucasians. It is reasonable to differentiate birth weight distributions by race only if race is itself a (non-pathological) determinant of fetal growth. Whether this is the case may be determined by comparing the estimations for optimal fetal growth, adjusted for non-pathological determinants, between populations of different races, after excluding pregnancies with evidence of exposure to pathological growth determining factors. If the estimations are significantly and systematically different, race specific standards are required. If they are not, the same standards for optimal fetal growth may be used even if the observed distributions in birth weight differ.

The aim of many of our exclusion criteria was to select a sample of births that had not been exposed to factors that have a pathological effect on intrauterine growth. Although the selection criteria only consider causes of growth anomaly we anticipated that selected births would be more likely to be born at term, be larger and born to taller, older and less disadvantaged mothers for several reasons. For example, pathologically affected growth is more often restricted than accelerated and is also associated with preterm birth and maternal smoking, the most prevalent pathological determinant of intrauterine growth, is associated with maternal age and socio-economic circumstances. Table 2 shows these anticipations to be realised. Selected births were also somewhat more likely to be female, supporting the general observation of the female advantage during gestation.

We believe that the curves shown in Figure 2 demonstrate that the exclusion of pathologically affected intrauterine growth, and of erroneous gestational estimates, has been reasonably successful. Biological growth (of which fetal growth is an example) typically proceeds to produce a Gaussian distribution at any point in time, with the standard deviation being proportional to the mean. Charts of unselected birth weights against gestational duration usually demonstrate increasing dispersion with decreasing gestation of delivery for deliveries before 40 weeks. There are two reasons for this: (i) the proportion of erroneously reported gestational age values increases with decreasing gestation, simply because the number of births actually delivered at any gestation week decreases the further it is from the modal value. Typically, erroneous preterm gestational reporting underestimates true gestational duration, hence the birth weight associated with an erroneously reported preterm gestational age is typically higher than those of births actually delivered at the reported gestation.

(ii) Birth much before term often has a pathological cause that also influences fetal growth. Thus the distributions of birth weights delivered at preterm gestations are no longer Gaussian, but typically, because pathological restriction occurs much more frequently than acceleration, are negatively skewed. Thus weights of neonates born at preterm gestations tend to be lower than fetuses of the same gestational age who go on to deliver at term.

In this study we address (i) by using the best estimate of gestational age that can be derived from all available data[16] and to exclude the '4-week errors' arising from gestational break-through bleeding. If we have succeeded in excluding pregnancies exposed to factors known to pathologically affect fetal growth we have addressed (ii), though the cost is the exclusion of a disproportionate number of infants born preterm, particularly very preterm, as can be seen in Table 2. The observation that the POBWs of the percentile positions are independent of gestational age, Figure 2, indicates that the dispersion is proportional to the mean across gestational age, and is compatible with both (i) and (ii) having been successfully addressed.

### Selection of independent variables

Some may consider our selection of predictor variables incomplete as it does not include measures of paternal size, maternal weight or maternal weight gain.

#### Paternal size

While paternal size is known to influence fetal growth it was not included because the biological father cannot routinely be identified and therefore measures of paternal size are not available on our database. The proportion of variability accounted for by the regression equations would, no doubt, be increased by the inclusion of paternal height as an independent variable.

#### Maternal weight

It has been suggested that maternal size affects fetal growth because it correlates with the area of uterine endometrium available for placentation. Since this area is not directly measurable, it is logical to seek and adjust for the maternal dimension(s) that correlates most closely with it. Maternal height measures skeletal size in the vertical dimension only, while maternal weight is associated with skeletal size and soft tissue mass, including adipose tissue. Skeletal height tends to correlate with skeletal size, but the proportion of weight consisting of soft tissue, particularly adipose tissue, is very variable, weakening the correlation between maternal weight and skeletal size. Data from the 5 month Dutch famine suggest that maternal pre-natal weight for height, a measure of soft tissue mass, is not a strong determinant of birth weight [25]. We therefore suggest that maternal height is likely to correlate better with the uterine area available for placentation than is prenatal maternal weight or weight for height.

#### Maternal weight gain

Maternal weight gain is occasionally considered to be a determinant of birth weight [26]. However fetal weight can be expected to correlate with maternal weight gain, because fetal weight, and its correlate placental weight, are significant components of maternal weight gain. Thus rather than being a non-pathological *determinant *of fetal weight, maternal weight gain partially *measures *fetal growth, whether or not it is optimal and should therefore not be adjusted for when estimating appropriateness of growth.

The non-pathological determinants of growth used in these models accounted for 40.5%, 32.2% and 25.2% of the variance in birth weight, length and head circumference respectively. The variation between these proportions may result from variation in the accuracy with which each birth dimension can be measured. Birth weight is routinely measured to within 5 g, representing about 0.15% of a median weight baby. Compared with birth weight birth, length is more difficult to measure reliably due to the tendency of the neonate to flex and the facility with which it may be stretched. Measured head circumference at delivery may be influenced by moulding of the head during passage through the birth canal. The effect of moulding on head circumference may be largely avoided by waiting until 2 days after birth before measurement. However with early discharge policies, such a wait risks failing to obtain any measurement of head circumference and in WA head circumference is routinely measured in the delivery room.

The highly significant, though small, dependence of head circumference on maternal age, despite adjustment for parity, was unexpected and requires confirmation in independent samples

#### Inclusion of maternal height and age as linear variables

All three dependent variables were found to have a linear dependence on maternal height in the range 147–183 cm. Outside this range there was a tendency for regression to the mean value of the dimension. This may occur because we could not include a term for paternal height and there will be a tendency for women at the extremes of height to have partners with heights that are less extreme. However since only 269 (~0.4%) of our selected sample had a height outside this range, maternal height was included as a linear variable.

Of the three dependent variables only head circumference had an association with maternal age, which was found to be linear up to age 45 years. Only 16 (0.03%) of our selected sample were older than 45 at the time of delivery, therefore maternal age was also included as a linear variable.

### Comparisons with previous methods of assessing intrauterine growth

In 1963 Lubchenco and colleagues [27] presented the first percentile charts of gender- and gestation-specific birth weights for an unselected population of live births and thereby initiated the modern study of intrauterine growth. The next major innovation in the methods of assessing intrauterine growth was the development of customised computer generated charts for individual neonates [4] by adding to gender and gestational duration the following predictor variables: maternal height, weight, ethnic group, parity and the birth weight, gestational duration and gender of any previous siblings, with the option of further adding measures of growth taken during the index pregnancy. The charts were again presented as percentiles. These charts were designed to predict birth weight rather than assess appropriateness of growth, as not all the independent variables (eg. sibling growth and ethnic group) are necessarily non-pathological determinants of intrauterine growth.

In 1993, Wilcox and colleagues [6] introduced the concept of the birth weight ratio, the ratio of the observed birth weight to the birth weight predicted given gestational duration, fetal gender, maternal height, weight, parity and ethnic group. Their study sample excluded multiple births, stillbirths and congenitally abnormal babies, and limited their analysis to term births, but did not attempt to exclude pregnancies affected by other pathological determinants of growth. Poor growth was defined on the basis of a percentile position of the birth weight ratio, thereby retaining the problems inherent in the use of percentile positions as standards.

## Summary

The method reported in this paper introduces two innovations, (i) using optimal, rather than expected, growth as the standard, and (ii) reporting the ratio of the observed to optimal birth dimension as the indicator of appropriateness of growth, rather than a percentile position.

We sought a sample with optimal opportunities for fetal growth for the creation of standard both because this is the logical standard and also to avoid the problem of the varying incidence of growth restricting pathology and environmental exposures between populations. Our previously published birth weight standards for Western Australia [17] excluded only perinatal deaths from the reference sample because other relevant data were not available at the population level. In subsequent models, we explored the possibility of using ratios rather than percentiles [11,20], the effects of maternal height and parity were estimated in broad strata and those of maternal age were not considered. Although births affected by some factors suggesting supoptimal growth could be excluded, data concerning the most commonly occurring pathological growth restricting exposure, maternal smoking, were not then available at the population level.

The creation of the standards for optimal fetal growth for Caucasian singletons presented here is possible in part due to additional methods of estimating gestational duration [16], the ability to exclude the large proportion of births to women who smoked or experienced factors known to affect fetal growth and more complete information concerning non-pathological determinants of growth. Computing and statistical methods have also been improved with the use of (a) the Box-Cox transformation to account for any non-normality in the distribution of the response variables (b) fractional polynomial regression which required no assumptions regarding the form of the relationship between gestational duration and each of the response variables and facilitated the use of continuous variables thereby allowing the effects of non-pathological determinants of growth to be estimated more precisely.

## Conclusion

We have presented a comprehensive guide to an alternative method of creating standards for newborn dimensions and assessing appropriateness of intrauterine growth. It is based on the estimation of the *optimal *value for the dimension which we define as the value obtained by regression techniques from a large sample of women without risk factors for intrauterine growth anomaly. In this method, appropriateness of intrauterine growth is expressed as the ratio of the observed birth dimension to the optimal birth dimension rather than as being above or below a specified percentile position of the population distribution of that dimension, avoiding the problems inherent in the use of percentile position. Since POBW is a **measure **of appropriateness of intrauterine growth it may be used as a continuous variable and subjected to parametric statistical analysis. The use of POBW in clinical and research settings will prove whether it is a more precise predictor of compromise within individuals than previously available indicators of intrauterine growth status.

## Abbreviations

GA: best available estimate of gestational age at delivery.

POBW: percentage of optimal birth weight.

WA: Western Australia.

## Competing interests

The author(s) declare that they have no competing interests.

## Authors' contributions

EB conceived of and directed the study and drafted the manuscript. YL carried out initial statistical analyses. NHdeK gave statistical advice. DML carried out subsequent statistical analyses.

All authors read and approved the final manuscript.

## Pre-publication history

The pre-publication history for this paper can be accessed here:


